# Mapping Health Needs to Support Health System Management in Poland

**DOI:** 10.3389/fpubh.2018.00082

**Published:** 2018-03-20

**Authors:** Tomasz Holecki, Piotr Romaniuk, Joanna Woźniak-Holecka, Adam R. Szromek, Magdalena Syrkiewicz-Świtała

**Affiliations:** ^1^Department of Health Economics and Health Management, School of Public Health in Bytom, Medical University of Silesia in Katowice, Bytom, Poland; ^2^Department of Health Policy, School of Public Health in Bytom, Medical University of Silesia in Katowice, Bytom, Poland; ^3^Department of Health Promotion, School of Public Health in Bytom, Medical University of Silesia in Katowice, Katowice, Poland; ^4^Department of Computer Science and Econometrics, Faculty of Organisation and Management, Silesian University of Technology, Gliwice, Poland

**Keywords:** health needs mapping, health care system, health management, Poland, health policy

## Abstract

In Poland, following the example of other EU countries, the first maps of health needs prepared by the Ministry of Health were presented in 2016. The maps constitute a foundation for rational decision-making in the management of health care resources, being potentially useful for all actors in health system. This refers in particular to the institutions responsible for distribution of funds and contracting health service, but also for decision-makers, who determine the scope of funds to be utilized in the health system, or the structure of benefits provided to patients. Service providers are also addressees of the maps, to give them a basis for planning future activities. The article presents a structured assessment of the current state of affairs, based on recent experience and sets out likely directions for the development of health needs in mapping in Poland in the future. We discuss the criticism addressed toward maps by representatives of various groups acting in health care. It includes the lack of recognition of some of the key health needs, or wrong emphases, where much more attention is paid to the recognition of current resources in the health system, instead of making prognoses regarding the future developments of health needs. Nonetheless, we find that this instrument is potentially of high usability, in case of elimination of the existing weaknesses.

## Introduction

Being responsible for the organization of health care systems, the European Union countries are trying to undertake a number of solutions to modify the performance and improve outcomes of their systems. These solutions include efforts to implement coordinated planning of treatment activities at the national and regional levels. One of the ways to rationalize existing procedures is to create, and then use in practice, the framework of health needs maps. In Poland public administration units are obliged to provide this kind of maps based on the provisions of Art. 95a and 95c of the Act of August 27, 2004, on health care services financed from public resources. Additionally there is a number of executive provisions to this Act, which confirm and specify this obligation (1, 2). First ever maps for Poland were prepared in 2016. The main body responsible for their publication was the Ministry of Health, which launched maps as part of a project cofinanced from the European Social Fund, under the operational program Knowledge, Education, and Development. As assumed by authors of the maps, they should become an instrument of improving the management of public funds in health care. The basic way of such improvement was to deliver data on current and projected health needs of patients, being subsequently mandatory used for planning decisional processes in health care (3, 4). Once the 16 regional and one national maps have been published, broad discussion emerged, and substantial criticism addressing the analytical tools used for the development of the maps has been presented. Although certain imperfections of the maps are somewhat obvious, due to the fact that this is the first project of this type implemented in Poland, it should be expected that they will be eliminated in subsequent releases.

According to the adopted assumptions, the first two editions of the maps are expected to be prepared by the Ministry of Health in cooperation with the regional branches of the National Health Fund, and in consultation with Voivodes—the regional representatives of the governmental administration. The map for the period from June 30, 2016, to December 31, 2018, was expected to be developed by April 1, 2016. The map for the period from January 1, 2019, to December 31, 2021, should be prepared by May 31, 2018. The first two editions of the maps are about to cover only hospital treatment. They are assumed to give background for preparing solutions to problems that will appear at the stage of their development. After 2021, projects of regional maps are to be published in 5 years intervals by the National Institute of Public Health—National Institute of Hygiene [Narodowy Instytut Zdrowia Publicznego-Państwowy Zakład Higieny (NIZP-PZH)], although they should be drawn up by regional governors (Voivodes) in consultation with the Regional Council for Health Needs. The drafts of maps should be delivered to NIZP-PZH, where a nationwide map will be developed, based on the content of regional maps. Once the maps are ready, the Minister of Health will approve and disseminate them (5).

## The Role and Purpose of Health Needs Maps

Health needs maps is an extensive analytical tool supporting managerial decisions in health care. They are aimed to present demographic and epidemiological trends, health care infrastructure and future needs in this area (6, 7). Additionally, maps may use a graphical projection to visualize all the included information in geographic dimension. Permanent implementation of this solution to the Polish health care system should help decision-makers to more effectively govern processes related to the allocation of physical, intellectual, and financial resources within the system (8, 9). The published maps are applicable to the entire Poland, as well as on the level of individual regions. They provide comprehensive information on demographic trends, health status of the population and available resources in the health system in terms of technical infrastructure and human resources. Information on resource utilization, namely hospital treatments, outpatient services in the areas of given specialties, and other services consumption, is also included in the maps. However, the essential part of the maps should be prognoses of future health needs, which is a derivate of the predicted future population structure, as well as projected incidence of given diseases determining the demand for specific medical services (10).

According to health care specialists, there are significant differences in health needs between individual regions, identified at the level of voivodships and poviats,^1^ which is a result of differences in health status of local populations. The role of health needs maps is therefore to identify these differences, and to give a background for planning interventions answering the most important and unmet health needs, as found in each administrative unit (11).

All participants of the health care market await this kind of source of information to be provided and to become available for their usage. Health services providers could obtain from the maps the data they need to minimize risks associated with making financial, human, and infrastructural investments. Patients and health services consumers might gain structured knowledge about medical potential in a given region, while the National Health Fund, which is the main payer in the system, should receive a rational foundation for long term planning of spendings, strictly connected with socioepidemiological reality (12). To achieve this, maps of health needs must include accurate records that include (10):

- length of queues and waiting times for ambulatory specialist services,
- length of queues and waiting times for outpatient diagnostic services,
- data on uncovered needs for treatment, particularly related to outpatient specialist treatment and hospital treatment,
- shortages in human resources, especially medical doctors of particular specialties, as well as other medical personnel, like nurses, psychologists, physiotherapists, dieticians, pharmacists, and other professionals,
- number of hospitalizations for the purpose of carrying out diagnostics that could be performed in the sector of outpatient care,
- availability of rehabilitation, palliative, and social care services,
- scope, quality, and effectiveness of preventive actions undertaken,
- hospital beds redundancy in relation to current needs.

Except of their prognostic dimension, health needs maps should provide also information about specific parameters to be basis for health system assessment, such as effectiveness, safety, consumer quality, accessibility, equality, efficiency, accessibility, comprehensiveness, continuity, as well as productivity, acceptance, and level of satisfaction among stakeholders of the health care system, and the dynamics of changes. The maps should become a foundation for the objective and correct diagnosis of the population’s health needs, along with providing possibility to make comparisons between regions, as well as between countries of the European Union. Maps may also enable institutions responsible for the governance of the health system to easily assess the effectiveness of individual health care organizations. In result, the processes of evaluation and planning should become possible to be controlled. Among other assumed benefits of using the maps, there is also a possibility of identifying areas, where the rate of return from the allocation of invested funds is highest. They also provide a basis for identification of areas requiring intervention in order to increase competitiveness and ensure financial stability. The maps should also provide a rationale for formulating targets for health system activities. Finally this instrument may help in reducing health inequalities, ensuring proper selection of priority interventions at the level of individual administrative units of different levels (6, 13, 14).

Maps may provide crucial information also for the planning of inpatient services, like annual bed occupancy rates. On this basis it is possible to evaluate the effectiveness of activities that has been provided, as well as to outline trends and seasonal fluctuations in the usage of hospital infrastructure in different sectors of treatment. Additionally, such data translated to population-standardized indicators will enable the supervisory bodies to assess the activities of medical entities in terms of the efficiency of resource usage, while maintaining appropriate safety standards and the quality of provided services. In case of outpatient specialist care, the maps will create a space for aggregating the data on the structure of units, usage of the services they provide, along with the number and types of diagnoses and the waiting time for the visit.

## Critical Analysis of Health Needs Maps in Poland

Despite all the assumed advances of the health needs maps, its first edition in Poland suffered important deficiencies. These defects were result of the time pressure during their development, as well as limited human resources engaged in their development. The defects of health needs maps are being outlined by health system stakeholders. According to the representatives of the Polish Oncological Society, their current edition published by the Ministry of Health confirm the existence of “white stains” in Polish oncology, when compared with international standards. This includes insufficiently equipped units providing radiotherapy, as well as low availability of current innovative pharmacological treatment. In their present form, the maps depreciate the role of oncological surgery as the leading diagnostic and therapeutic method. The maps also insufficiently outline the perspective of the development of histopathological and molecular diagnostics. At the same time, in the Society’s opinion, from the perspective of strategic planning the development of oncology, a priority should be given to efforts to ensure equal access to specialized combined treatment for all patients, instead of analyzing the availability of individual oncological treatment methods. Except of that, it is fundamentally important to establish a network of certified medical facilities evenly distributed in the territory of the country, to provide quick oncological diagnostics. Finally, the maps should also outline the growing role of primary care physicians in the field of oncological prevention and diagnosis (15).

Associations of patients in turn, point out that the health needs maps should focus to a much greater extent on prediction of the most important health needs of patients. In their present form they are more concentrated on the identification of current state of things in health care. On the other hand, while making the current diagnosis, the maps should also include accurate records and analysis of waiting times for outpatient specialist care and diagnostic examinations. They should also clearly define existing shortages in terms of medical professionals, namely physicians of particular specialties, nurses, psychologists, physiotherapists, dietitians, pharmacists, etc. Finally, the maps should address issues of accessibility to rehabilitation, palliative, and social care services (16).

Critics point out that maps of health needs should anticipate the state of resources in the health care system and that they should be regularly adjusted to the dynamics of the health system reality, including changes in the health needs of the population. Meanwhile, a critical part of the existing study is just an inventory of the current range of services provided in individual voivodships, constructed based on an overview of the registers kept by the health care entities and National Health Fund. This makes the maps to be *de facto* nothing more, but a simplified product analysis. Critics of the maps also pay attention to the fact that they ignore the limitations in availability of services resulting of the public payer policy, due to budget constraints. Finally the maps focus only on in-patient care, while omitting ambulatory services, prevention, coordination of services, and the improvement in quality, effectiveness, and safety of treatment that are being achieved in this sector (16, 17).

The organizations of physicians’ self-government declared that they agree with the necessity of implementing instruments for the health care planning based on the analysis of existing resources and health needs. Nonetheless, the presidium of the Polish Chamber of Physicians and Dentists, having considered the scope of health needs maps, postulated, to amend the project in several different aspects. According to medical self-government officials, the scope of maps should include also data on the migration rates in regions. Beside of that, the maps should be also enhanced with broader outlook, including the public health perspective (18).

Thus, many experts active in the field of health care, including oncology in particular, presented their critical remarks about the procedure of development and content of partial reports, of which the current health needs maps consist of. One of the crucial remarks is to draw attention to interregional migrations and the related necessity to adapt the resources of the health care system to dynamically changing demographic status of regions. However, the most important thing is to make the maps a future-oriented management tool for health care, not just a retrospective analysis of facts. This is all the more important, that the currently available epidemiological and demographic data makes the predictions of trends possible to be obtained, which is not always easy in the field of medical sciences. The suggested way of improving the maps may be a contribution to the amelioration of the health care system management in Poland.

## Concluding Remarks and Recommendations

As assumed, all decisions in the Polish health care system that are going to be made after publication of the health needs maps should be based on the information included therein. This includes justifications for making individual investment decisions by health care institutions and local public administration units. The National Health Fund is expected to assess the scope and number of contracted procedures based on the information retrieved from the maps. In order to strengthen the rationality of the decision-making processes the maps are expected to be used at all decisional levels in the health care system: national (Ministry of Health, National Health Fund), regional (voivodships), local (poviats, gminas—both in terms of providing their public health tasks for local communities, and as owners of health care facilities), and on the level of individual health care institutions managerial bodies. This means that institutions that are responsible for preparation of merit-based health needs maps have been burdened with a demanding and responsible task, that is to create an actual foundation for all fundamental decisions in health care. The success in their work will subsequently determine the effectiveness of future decision-making processes, while the failure or defectiveness of the maps may make all efforts to improve the efficiency of the system in vain. Unfortunately, the current shape of the maps is not satisfying. It should be expected that future releases will gradually be deprived of current imperfections. If this happens, the revised and structured health needs maps have a chance to become a solid foundation for shaping a rational health policy in Poland. In particular, they should be improved in accordance with the suggestions of bodies and stakeholders, for whom the maps constitute a long-awaited supportive tool in the decision-making processes they are initiating, or that are affecting them. If the health needs maps are expected to be an adequate instrument for planning health policy and health care management, there are few crucial elements that must not be omitted in their subsequent releases:

- a more thorough analysis of the demographic context specifying trends in the structure and size of populations of individual regions, in connection with
- definitely more prospective picture of the health needs, so that the managerial decisions regarding technical infrastructure, professionals, and also the financial resources necessary to be engaged in the provision of health care services could be planned in advance, in a manner adequate to the expected epidemiological and social situation.
- Additionally, it should be stressed that the implementation of activities within the health system should remain in close connection with other areas of state’s interventions in social policy. In case of planning specific policies to modify unfavorable demographic trends, like implementation of programs to increase fertility or various aspects of migration policy, the infrastructural and human resources of the health care system should be shaped in a way as to ensure effective achievement of assumed aims in different sectors of social policy. At the same time, investment decisions and other managerial procedures in health care must not be a passive response to processes occurring in a spontaneous manner, but they should anticipate the state of affairs achieved in result of active stimulation of demographic processes by the national policies.
- Finally, except of outpatient and hospital services, also other categories of health services should be a subject for projecting the demand. The example might be spa treatment, which is an immanent part of health system in Poland, strongly embedded in its tradition and system of preventive and curative resources. In context of expected process of population aging, this category of services should be expected to even increase its relevance in the catalog of health needs (19).

## Author Contributions

TH prepared the draft of the article and contributed to study design and main thesis. PR prepared the final version of the article and contributed to data collection, study design, and main thesis. JW-H collected data and contributed to the article draft. AS prepared the article main thesis and designed the study. He contributed to the article draft. MS-S contributed to the article draft and data collection.

## Conflict of Interest Statement

The authors declare that the research was conducted in the absence of any commercial or financial relationships that could be construed as a potential conflict of interest.

## Footnotes

^1^Polish administrative structure includes three levels of local administration: voivodeships (województwa—regional level units), poviats (powiaty—local middle-level units), and gminas (gminy—cities, towns, and communities; the basic level). Gminas and poviats are self-governing units, while voivodeships are mixed self-governing and government-dependent units. Regional Legislative (Sejmik) is being elected by community living in the region, and then subsequently appoints an executive body (Marszałek—Marshall), while Voivode (wojewoda, regional governor) is being appointed by the national government.
